# PP5 (PPP5C) is a phosphatase of Dvl2

**DOI:** 10.1038/s41598-018-21124-3

**Published:** 2018-02-09

**Authors:** Jianlei Xie, Meng Han, Miaojun Zhang, Haiteng Deng, Wei Wu

**Affiliations:** 1 MOE Key Laboratory of Protein Science, School of Life Sciences, Tsinghua University, Beijing, 100084 China; 2 MOE Key Laboratory of Bioinformatics, School of Life Sciences, Tsinghua University, Beijing, 100084 China

## Abstract

Dishevelled (Dvl) family proteins are key mediators of Wnt signalling and function in both canonical and noncanonical branches. Dvl2, the most studied Dvl protein, is extensively regulated by phosphorylation. Several kinases were found to be critical for Dvl2 localisation, stability control and functional segregation. For example, S143-phosphorylated Dvl2 was detected, together with CK1δ/ε, at the centrosome and basal body of primary cilia and plays pivotal roles during ciliogenesis. However, relatively less is known about Dvl dephosphorylation and the phosphatases involved. Here, we identified PP5 (PPP5C) as a phosphatase of Dvl2. PP5 interacts with and can directly dephosphorylate Dvl2. Knockdown of PP5 caused elevated Dvl2 phosphorylation both at the basal level and upon Wnt stimulation. In the Dvl2 protein, S143, the 10B5 cluster and other sites were dephosphorylated by PP5. Interestingly, comparison of PP5 with PP2A, another known Dvl2 phosphatase, revealed that PP5 and PP2A are not fully redundant in the regulation of Dvl2 phosphorylation status. In hTERT-RPE1 cells, PP5 was found at the basal body of cilia, where S143-phosphorylated Dvl2 also resides. Functional assays revealed modest effects on ciliogenesis after PP5 depletion or over-expression. Taken together, our results provided evidence to suggest PP5 as a new phosphatase for Dvl2.

## Introduction

Dishevelled (Dvl) is a cytoplasmic adaptor protein necessary for Wnt signalling and functions in both canonical and noncanonical signalling branches^1,2^. Through Dvl family proteins, canonical Wnt signalling regulates cell proliferation and cell fate decision^3,4^, whereas noncanonical Wnt signalling controls cell polarity, cell migration and some other events such as centrosome positioning and primary ciliogenesis^5–7^. Dvl consists of an N-terminal DIX domain, a central PDZ domain and a C-terminal DEP domain. Generally, DIX and PDZ domains mediate functions in canonical Wnt signalling, while PDZ and DEP domains are required for noncanonical signalling^1^. As an adaptor protein, the signalling functions of Dvl are achieved through interactions with many other proteins. Upon canonical Wnt ligand stimulation, Frizzled (Fz) receptors interact with the PDZ domain to recruit Dvl to the cell membrane for signal activation^8^. DIX domain mediates the interaction with Axin and also participates in its membrane recruitment^9–11^. PDZ domain and DEP domain together activate the RhoA/Rac family of small GTPases in noncanonical Wnt signalling^12,13^. Sequences between the three domains and of the C-terminus are also functional in signal transduction under certain conditions^14–16^. There are three homologues of Dvl in humans: Dvl1, Dvl2 and Dvl3. Although functional specialization is apparent, they share similar structures and types of regulation in many contexts^17–20^.

At the cellular level, the function of Dvl proteins is critically regulated by phosphorylation^1,2^. Upon Wnt ligand stimulation, Dvl proteins are rapidly and intensely phosphorylated^15,21,22^. Several kinases have been shown to phosphorylate Dvl, such as CK1δ/ε, CK2, PAR-1, Abl, RIPK4 and NEK2^23–30^. More than 50 phosphorylation sites have been identified in Dvl proteins, most of which are serine/threonine residues^16^. The functional significance of phosphorylation at several sites has been investigated in detail. For instance, S143 and T224 sites of human Dvl2 were shown to be phosphorylated by CK1δ/ε. This creates an interface for Plk1 interaction. The resulting Dvl2–Plk1 complex promotes HEF1 stabilisation and then Aurora A activation at the basal body of primary cilia. This process is required for the serum-stimulation-induced disassembly of primary cilia in human retinal pigmented epithelial cells (hTERT-RPE1)^6^. It was also reported that CK1ε phosphorylates S594, T595, S597 and T604 sites of human Dvl2 in response to Wnt stimulation. The phosphorylation of this cluster reduced the recognition by a monoclonal Dvl2 antibody, 10B5. In addition, mutation of these sites (10B5 sites hereafter) into alanine led to increased punctate localisation and canonical Wnt signal activation^15^.

Considering the importance of the phosphorylation of Dvl proteins, several Dvl phosphatases have also been identified and functionally investigated. The catalytic subunit of PP2A (PP2A/C) binds directly to the DEP domain to dephosphorylate Dvl2. Wnt3a treatment increases this interaction but decreases the phosphatase activity of PP2A/C^31^. The catalytic subunit of PP1 (PP1c) was also reported to be a Dvl phosphatase. PP1c dephosphorylates Dvl with the aid of Hipk2, and relieves Dvl from Itch-mediated ubiquitination. This process results in the stabilisation of Dvl and maintains the normal Dvl protein level for Wnt signal transduction in mammalian cells and zebrafish embryos^32^. Interestingly, Dvl is also a protein substrate of PTEN. The interaction with PTEN is mediated by the DEP domain and the C-terminus of Dvl2. PTEN dephosphorylates the S143 site of Dvl2 and regulates the disassembly of primary cilia in hTERT-RPE1 cells^7^.

PP5 (PPP5C) is a serine/threonine phosphatase of the phosphoprotein phosphatase (PPP) family^33–35^. Unlike most other PPP family phosphatases, which form holoenzymes with a great number of regulatory subunits to recognise substrates, PP5 is a single-subunit enzyme and utilises its N-terminal TPR domain to achieve substrate recognition and activity regulation^36–38^. The basal phosphatase activity of PP5 is relatively low as a result of the intramolecular autoinhibition achieved through the interaction between the TPR domain and a C-terminal αJ helix^39,40^. Interaction of the TPR domain with Hsp70/Hsp90 relieves the autoinhibition of PP5^40–43^. Functionally, PP5 has been proved to be an important regulator of hormone and stress-related signalling^33,34^.

Here, we identified PP5 as a phosphatase of Dvl2. PP5 interacts with and dephosphorylates Dvl2. CK1ε was shown to promote the interaction between PP5 and Dvl2. Knockdown of PP5 led to increased Dvl2 phosphorylation in response to both Wnt3a and Wnt5a treatment. Both S143 and 10B5 sites of Dvl2 were shown to be dephosphorylated by PP5. Moreover, coexpression with constitutively active PP5 mutants promoted punctuate localisation of Dvl2. Conversely, overexpression of the TPR domain, likely via its dominant negative effect, inhibited the dephosphorylation of Dvl2 by PP5 and resulted in diffuse cytoplasmic localisation of Dvl2. In hTERT-RPE1 cells, PP5 was found at the basal body of primary cilia, where S143-phosphorylated Dvl2 was also detected. Over-expression or depletion of PP5 moderately affected ciliogenesis, possibly through dephosphorylating the S143 site of Dvl2. Taken together, our results show that PP5 is a phosphatase of Dvl2.

## Results

### Identification of Dvl2 phosphatase

In searching for phosphatases involved in Dvl2 dephosphorylation, we coexpressed FLAG-Dvl2 in HEK293T cells together with plasmids from a library containing 106 clones encoding 96 phosphatases or related genes (Table S1). The mobility shift of the Dvl2 protein in SDS-PAGE was used as an indicator of its phosphorylation status. The appearance of more rapidly migrating bands indicated the dephosphorylation of FLAG-Dvl2 by coexpressed genes. Four phosphatases, namely, PPP2CA, PPP2CB, PPP5C and PPM1A, encoded by five clones were identified (Fig. S1). PPP2CA and PPP2CB encode catalytic subunits of PP2A, which is a well-known Dvl2 phosphatase^31^. PPM1A encodes a metal-dependent protein phosphatase of the PP2C family^44^, which has already been reported to interact with Dvl2 and dephosphorylate Axin in regulating Wnt signalling^45^. The identification of known Dvl2 phosphatases indicated that the screen was sufficiently sensitive. PPP5C encodes protein phosphatase 5 (PP5), which has been shown to regulate several signalling pathways^33,34^. However, to our knowledge, no direct link between PP5 and Dvl has been suggested. Therefore, we focused on PP5 to investigate its effects on Dvl2 dephosphorylation and function.

### PP5 can dephosphorylate Dvl2

**Figure 1:**
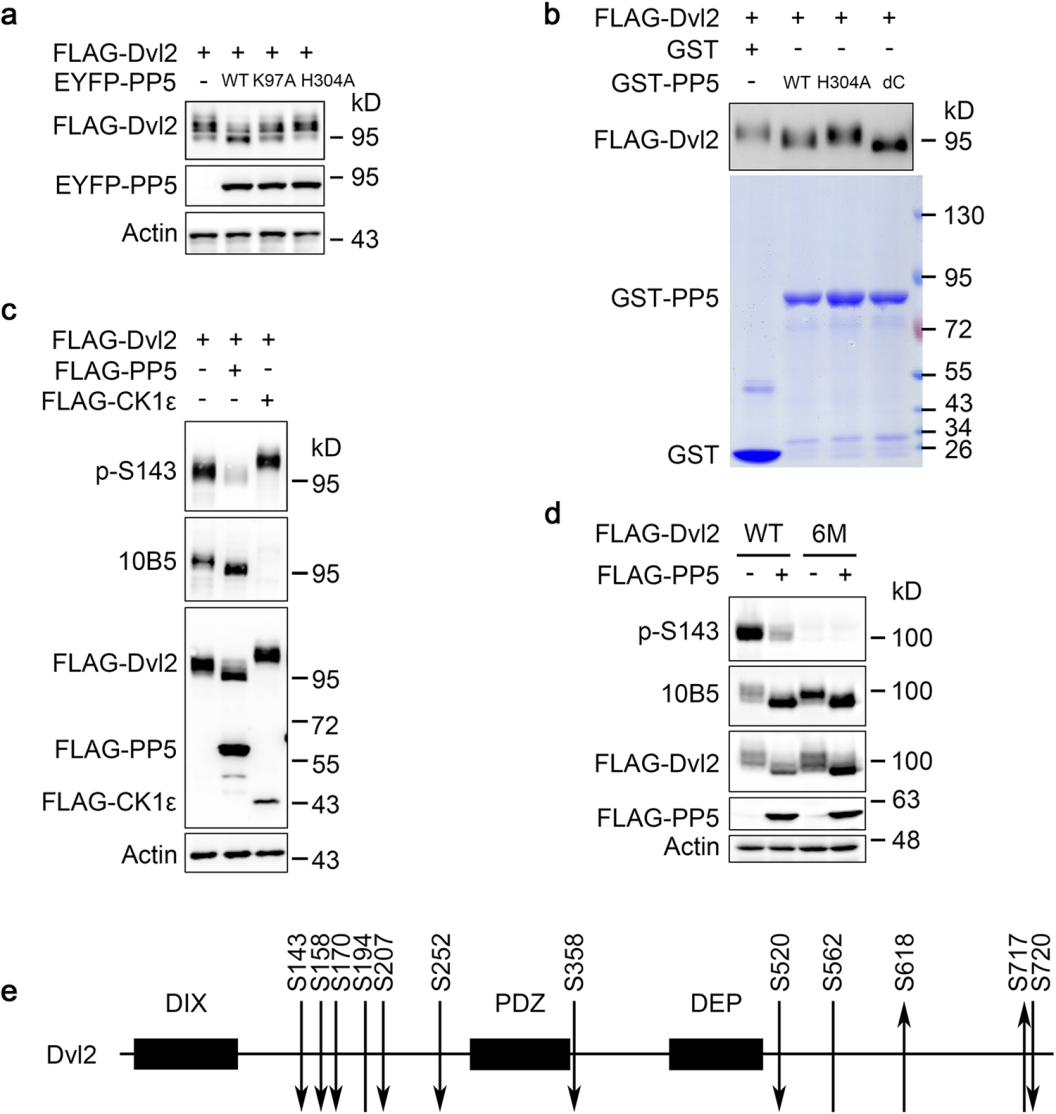
PP5 dephosphorylates Dvl2. (**a**) HEK293T cells were transfected with FLAG-Dvl2 together with wild-type or mutant PP5 and analysed by Western blotting (WB) with the indicated antibodies. (**b**) FLAG-Dvl2 purified from transfected HEK293T cells was incubated with PP5 or PP5 mutant proteins purified from *E. coli* in phosphatase assay buffer for 1 h at 30 °C. GST was used as a negative control. The protein samples were then subjected to WB to verify Dvl2 phosphorylation. (**c**) HEK293T cells were transfected with FLAG-Dvl2 together with FLAG-PP5 or FLAG-CK1ε and then analysed by WB with the indicated antibodies. Note that p-S143 antibody recognises Dvl2 with S143 phosphorylation, while 10B5 antibody recognises Dvl2 with its 10B5 sites not phosphorylated. (**d**) HEK293T cells were transfected and analysed by WB. WT, wild-type Dvl2. 6 M, Dvl2 S143A/T224A/S594A/T595A/S597A/T604A mutant. (**e**) Schematic showing the Dvl2 phosphorylation sites identified by MS analysis with their change of phosphorylation level indicated by arrows. Downward arrow indicates more than twofold reduction by PP5. Upward arrow indicates more than twofold upregulation by PP5. Line without an arrowhead indicates no significant change detected. Full-length gels and blots of this figure are shown in Figure S4.

To confirm that PP5 can dephosphorylate Dvl2, we coexpressed FLAG-Dvl2 together with wild-type PP5, PP5-K97A, a less active mutant, or PP5-H304A, a phosphatase-dead mutant, in HEK293T cells. As shown in Fig. 1a, wild-type PP5 caused strong fast migrating bands of Dvl2 in SDS-PAGE, whereas PP5-K97A caused a partial shift and PP5-H304A caused none. Next, we purified FLAG-tagged Dvl2 protein from transiently transfected HEK293T cells and incubated it with bacterially expressed and purified PP5 proteins (GST-PP5). Wild-type PP5 protein directly dephosphorylated Dvl2, indicated by a clear down-shift of the Dvl2 bands (Fig. 1b). PP5-H304A mutant did not dephosphorylate Dvl2. Interestingly, PP5-deltaC (PP5-dC), which lacks amino acids critical for its C-terminal inhibitory domain and thus is believed to be a constitutively active form^39^, was indeed highly active in promoting the rapid migration of Dvl2 (Fig. 1b).

Although mobility shift has been widely used as an assay of Dvl2 phosphorylation, we made use of two antibodies to further investigate the phosphorylation sites in Dvl2. The first one is an antibody recognising the phosphorylated serine 143 site of human Dvl2 (p-S143). The second one (clone 10B5) reacts with human Dvl2 with its four C-terminal serine/threonine sites (S594, T595, S597 and T604; collectively called 10B5 sites) unphosphorylated^15^. Thus, a signal from the 10B5 antibody indicates the loss of phosphorylation at these sites, and loss of the 10B5 signal indicates that these sites are phosphorylated^15^. Upon PP5 coexpression, the p-S143 signal was largely abolished and the 10B5 signal was enhanced (both representing Dvl2 dephosphorylation), accompanied by a dramatic down-shift of FLAG-Dvl2 protein (Fig. 1c). In contrast, CK1ε coexpression caused slow-migrating (up-shift) FLAG-Dvl2 proteins and complete abolition of the 10B5 signal. The p-S143 signal was not significantly enhanced by CK1ε, probably because Dvl2 from HEK293T cells was already highly phosphorylated on S143. These results indicate that PP5 can at least dephosphorylate the S143 and 10B5 regions of the Dvl2 protein.

Dvl2 harbours many more phosphorylation sites other than the S143 and 10B5 region^1^. To investigate whether there are other sites in Dvl2 that are dephosphorylated by PP5, we mutated S143, T224 and the 10B5 sites (S594, T595, S597 and T604) of human Dvl2 all into alanine. The mutated protein (Dvl2-6M) migrated more rapidly in gels than the wild-type protein (WT), indicating less phosphorylation. However, coexpression with PP5 caused a further down-shift of Dvl2-6M protein (Fig. 1d). This result suggested that PP5 can also dephosphorylate some other sites of Dvl2. To clarify this issue, mass spectrometry (MS) was used to identify potential sites affected by the coexpressed PP5. EYFP-Dvl2 was expressed alone or together with FLAG-PP5 in HEK293T cells and Dvl2 protein was purified and analysed (Fig. S2a). Twelve phosphorylation sites of Dvl2 were identified by mass spectrometry (Fig. 1e). Coexpression with PP5 led to the significant dephosphorylation of eight sites (S143, S158, S170, S207, S252, S358, S520 and S720). The phosphorylation status of two sites (S194 and S562) was not significantly changed by PP5. Besides, the phosphorylation status of the other two sites (S618 and S717) was significantly increased upon coexpression with PP5 (Figs 1e, S2b, Table S2). Taking these findings together, PP5 can dephosphorylate multiple sites of the Dvl2 protein.

### PP5 interacts with Dvl2

**Figure 2:**
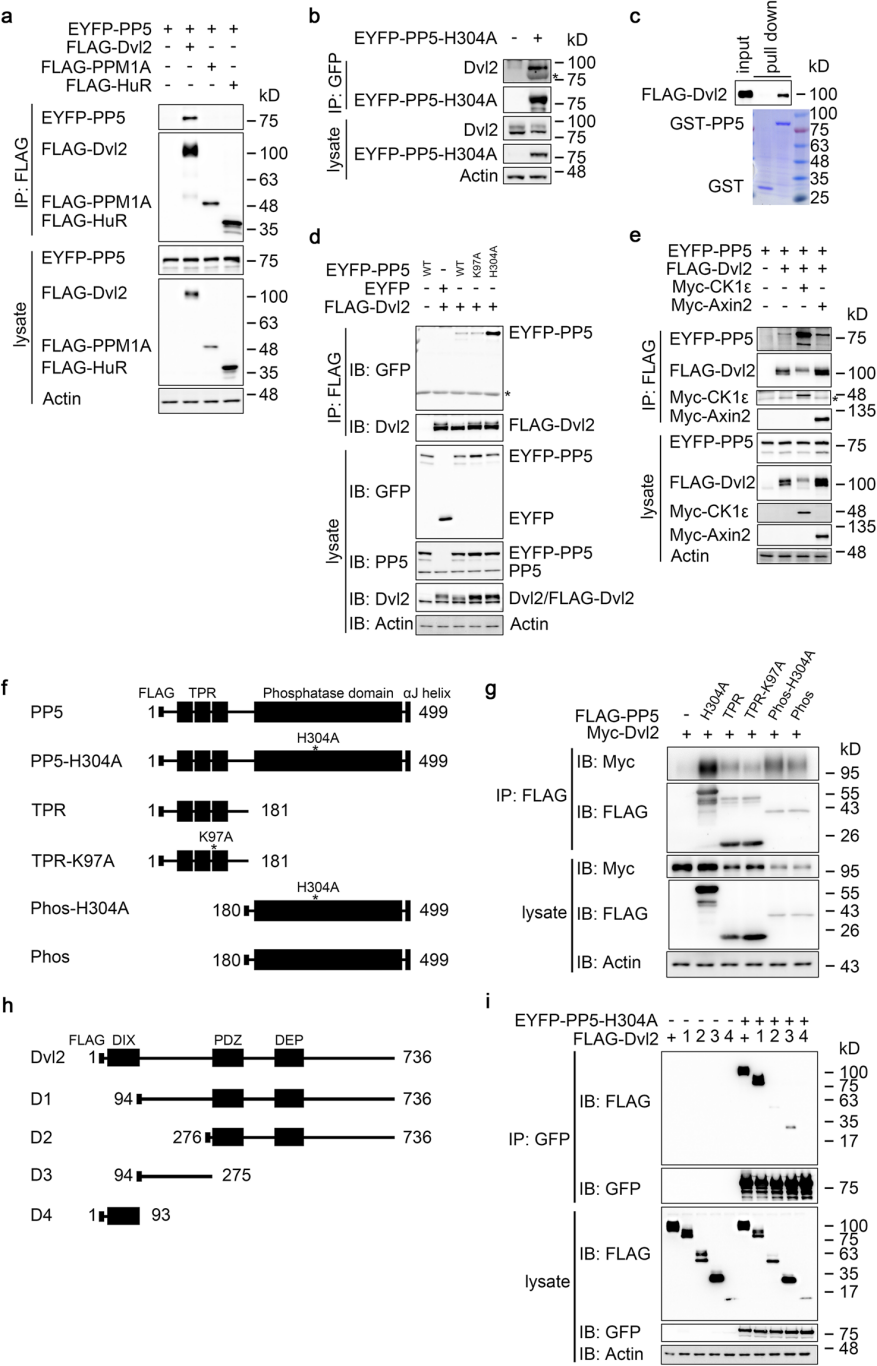
PP5 interacts with Dvl2. (**a**) Transfected HEK293T cells were processed for coimmunoprecipitation with FLAG M2 beads and samples were analysed by WB. (**b**) HEK293T cells were transfected as indicated and processed for Co-IP with GFP-Trap beads. Asterisk indicates nonspecific signal from enriched EYFP-PP5-H304A protein. (**c**) FLAG-Dvl2 purified from transfected HEK293T cells was incubated with PP5 protein purified from *E. coli* together with Glutathione Sepharose 4B beads. GST was used as a negative control. (**d**,**e**) HEK293T cells were transfected as indicated and processed for Co-IP with FLAG M2 beads. WT, wild-type PP5. K97A, PP5-K97A. H304A, PP5-H304A. Asterisk indicates nonspecific bands. (**f**) Schematic of PP5 mutants used in (**g**). (**g**) HEK293T cells were transfected as indicated and processed for Co-IP with FLAG M2 beads. Samples were then analysed by WB. (**h**) Schematic of Dvl2 truncations used in (**i**). (**i**) HEK293T cells were transfected with EYFP-PP5-H304A and FLAG-tagged full-length or truncated Dvl2. Cells were processed for Co-IP with GFP-Trap beads and were then subjected to WB. Full-length blots of this figure are shown in Figure S5.

PP5 consists of an N-terminal TPR domain and a C-terminal phosphatase domain^36–38^. The TPR domain has been suggested to function in protein-protein interactions^42,46^. It is also bound by a short amino acid sequence from the C-terminus of PP5 protein and this interaction functions as an autoinhibitory mechanism^40^. Hsp90, via its interaction with the TPR domain, releases this autoinhibition^40^. To determine whether PP5 interacts with Dvl2, Co-immunoprecipitation (Co-IP) experiments were carried out in HEK293T cells. The results indicated that PP5 was effectively co-immunoprecipitated with Dvl2, while no interaction was detected with other unrelated proteins (Fig. 2a). A semi-endogenous Co-IP experiment was conducted and the endogenous Dvl2 was indeed associated with the transiently expressed phosphatase-dead PP5 (PP5-H304A) (Fig. 2b). Moreover, GST-pull down assay confirmed that PP5 binds to Dvl2 directly (Fig. 2c). In comparison with the wild-type PP5, PP5-K97A, which contains a mutation in the TPR domain that disrupts the interaction of PP5 with Hsp90, showed similar interaction with Dvl2. Interestingly, the phosphatase-dead mutant PP5-H304A showed much stronger interaction with Dvl2 (Fig. 2d). In this experiment, the levels of transiently expressed FLAG-Dvl2 and EYFP-PP5 were not much higher than those of their endogenous counterparts, suggesting that the interaction observed is physiologically relevant (Fig. 2d). Since coexpressed PP5 significantly reduced the overall phosphorylation of the Dvl2 protein, while the phosphatase-dead form did not, we tested whether elevated phosphorylation on Dvl2 could enhance PP5 binding. Indeed, coexpression with CK1ε led to increased Dvl2 phosphorylation (upper shift), accompanied by increased affinity to PP5. Coexpression of Axin2, another well-known Dvl2 interacting protein, did not affect the interaction between PP5 and Dvl2 (Fig. 2e). These results suggest that PP5 interacts with Dvl2 and this interaction is further enhanced by Dvl2 phosphorylation.

To investigate which part of PP5 is involved in Dvl2 interaction, four additional constructs were prepared, namely, TPR domain alone (TPR), TPR-K97A in which the TPR domain contains the K97A mutation, phosphatase domain alone (Phos) and Phos-H304A in which the phosphatase domain is inactive (Fig. 2f). Phosphatase-dead full-length PP5 (PP5-H304A) was used as a positive control in this Co-IP experiment (Fig. 2g). The results indicated that both TPR and phosphatase domains were involved in the interaction with Dvl2. Compared with the phosphatase domain (Phos), Phos-H304A mutant showed a stronger interaction (Fig. 2g).

Next, the domain of Dvl2 that interacts with PP5 was determined (Fig. 2h,i). Full-length Dvl2 interacted strongly with PP5-H304A and the deletion of the DIX domain (D1) did not affect their interaction. However, the D2 mutant, which lacks all N-terminal sequence from the PDZ domain, exhibited a dramatic decrease in its interaction with PP5-H304A. Therefore, the sequence between DIX and PDZ domains seems to be essential for the interaction. In fact, this region alone (D3) was sufficient for the interaction with PP5-H304A to occur, albeit with relatively low affinity (Fig. 2i). Of course it is possible that some flanking sequence or sequences in other parts are also involved.

### PP5 is required for Dvl2 dephosphorylation

**Figure 3:**
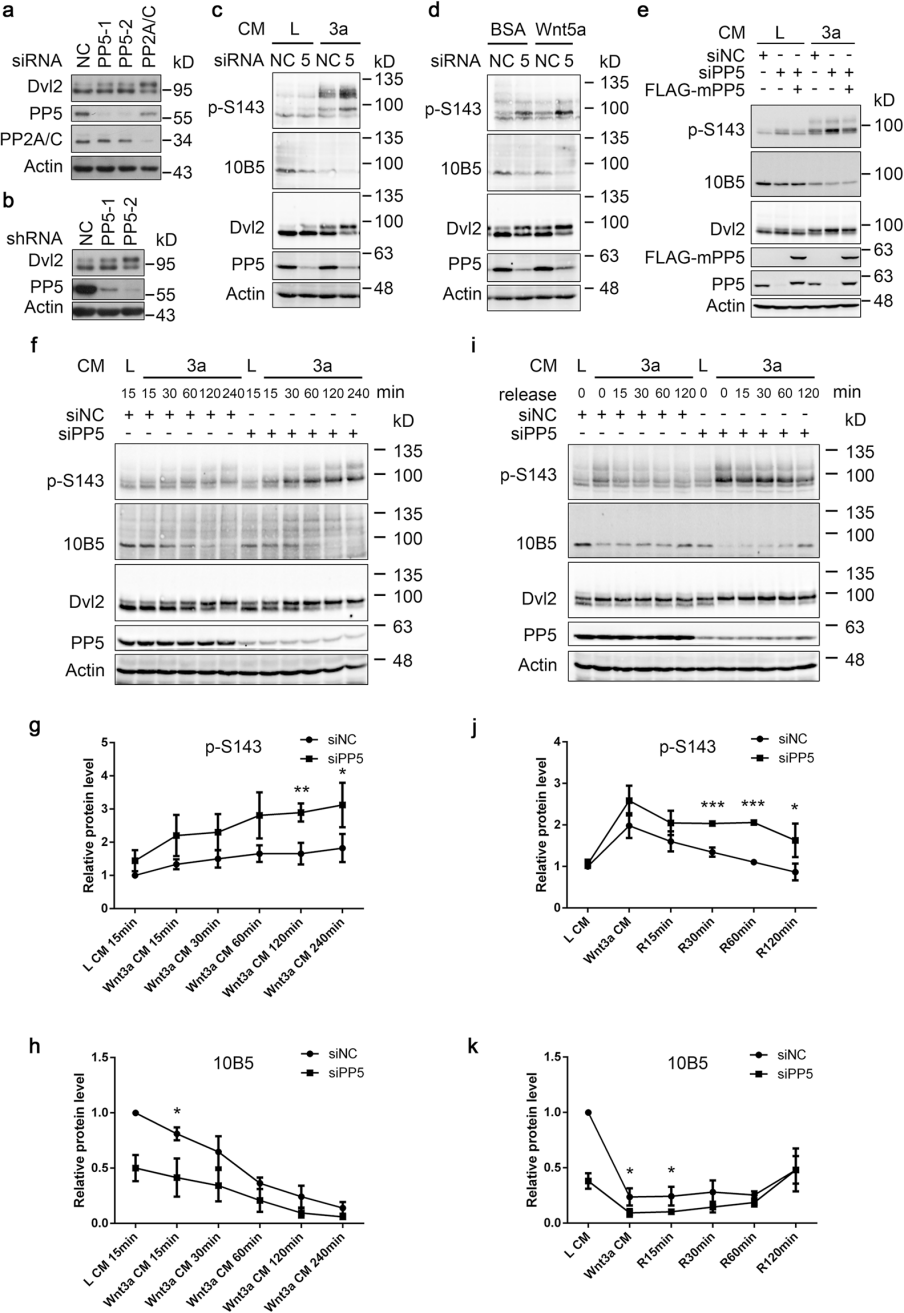
PP5 is required for Dvl2 dephosphorylation. (**a**) MCF7 cells were transfected with two independent PP5 siRNAs. Cells were lysed 48 h later and analysed by WB. siRNA targeting PP2A/C was used as a positive control. NC, negative control siRNA. (**b**) HCT116 cells stably expressing two independent shRNAs targeting PP5 were lysed and analysed. NC, negative control shRNA. (**c**,**d**) HCT116 cells were transfected with control or PP5 siRNA and 48 h later, cells were treated with Wnt3a conditioned medium (CM) (**c**) or Wnt5a protein (**d**) for 3.5 h. Cell lysates were analysed by WB. L CM and BSA were used as a control. L, L CM (conditioned medium collected from control L-cells). 3a, Wnt3a CM. NC, negative control siRNA. 5, PP5 siRNA. (**e**) HCT116 cells were transfected with indicated siRNA and 24 h later, cells were further transfected with plasmids as indicated. After another 24 h, cells were treated with L or Wnt3a CM for 2 h. L, L CM (conditioned medium collected from control L-cells). 3a, Wnt3a CM. (**f**) HCT116 cells were transfected with control or PP5 siRNA and 48 h later, cells were treated with L or Wnt3a CM for the indicated time periods. L, L CM (conditioned medium collected from control L-cells). 3a, Wnt3a CM. (**g**,**h**) Quantification of p-S143 (**g**) and 10B5 (**h**) signal intensity from (**f**). The intensities of the phosphorylation signal and total Dvl2 signal from each sample were independently quantified and their ratio was calculated. The ratio from L-CM-treated, siNC-transfected cells was set as 1 and was used to normalise the other samples. Results from three independent experiments were used for statistical analysis. Error bars, S.D. *p < 0.05, **p < 0.01 (*t*-test). (**i**) HCT116 cells were transfected with control or PP5 siRNA and 48 h later, the cells were treated with L CM or Wnt3a CM for 2 h to stimulate Dvl2 phosphorylation. Then, cells were washed with PBS and cultured in normal medium for the indicated time periods before harvesting and analysis. L, L CM (conditioned medium collected from control L-cells). 3a, Wnt3a CM. (**j**,**k**) Quantification of p-S143 (**j**) and 10B5 (**k**) signal intensity from (**i**). The intensities of the phosphorylation signal and total Dvl2 signal from each sample were independently quantified and their ratio was calculated. The ratio from L-CM-treated, siNC-transfected cells was set as 1 and was used to normalise the other samples. Results from three independent experiments were used for quantification. Error bars, S.D.*p < 0.05, ***p < 0.001 (*t*-test). Full-length blots of this figure are shown in Figure S6.

The above results indicate that PP5 can interact with and dephosphorylate Dvl2. To investigate whether PP5 is required for Dvl2 dephosphorylation, we used two independent siRNAs to knockdown PP5 in MCF7 (Fig. 3a), HCT116, HEK293T and HeLa cells (data not shown). The PP5 protein level was efficiently downregulated upon siRNA transfection. At the same time, the phosphorylation level of endogenous Dvl2, judged by the enhanced intensity of the upper band, was increased in cells with PP5 knockdown (Fig. 3a). As a positive control, knocking down PP2A/C (a catalytic subunit of PP2A) also led to an increase in Dvl2 phosphorylation (Fig. 3a). Moreover, two independent shRNAs were used to knockdown PP5 in HCT116 cells, and elevated Dvl2 phosphorylation was also observed (Fig. 3b).

Dvl2 phosphorylation can be further stimulated by Wnt ligands of both canonical and noncanonical signalling^15,21,22^. We therefore treated PP5 knockdown HCT116 cells with Wnt3a conditioned medium (CM) or Wnt5a protein and verified Dvl2 phosphorylation. Wnt ligands stimulated Dvl2 phosphorylation in both S143 and 10B5 sites, and PP5 knockdown further enhanced them (Fig. 3c,d). The effects of PP5 knockdown were largely rescued by coexpression of mouse PP5, which was not effectively targeted by the siRNA (Fig. 3e). To shed light on the dynamic process, HCT116 cells were treated with Wnt3a CM for different time periods and the Dvl2 phosphorylation level was quantified (Fig. 3f–h). PP5 knockdown resulted in a significant increase of Dvl2 phosphorylation in both S143 and 10B5 sites in the whole process of 4-h Wnt3a treatment. These results demonstrate that PP5 is involved in the regulation of both the basal level and Wnt-ligand-induced Dvl2 phosphorylation.

We also attempted to clarify the effects of PP5 knockdown on the process of active Dvl2 dephosphorylation. For this purpose, HCT116 cells were treated with Wnt3a CM for 2 h to stimulate Dvl2 phosphorylation. After removing Wnt3a CM, cells were washed with PBS and supplemented with normal medium to allow the dephosphorylation of Dvl2 (Fig. 3i–k). In control siRNA-transfected cells, S143 phosphorylation (p-S143) was soon diminished after the removal of Wnt3a CM. However, in PP5 knockdown cells, the existence of a high p-S143 level was significantly prolonged (Fig. 3i,j). Interestingly, the recovery of the 10B5 signal, that is, the dephosphorylation of 10B5 sites, was relatively slow and knockdown of PP5 only slightly prolonged this process (Fig. 3i,k). Similar results were also obtained from HCT116 cells in which PP5 was downregulated by shRNAs (data not shown). The above results indicate that PP5 is involved in the dephosphorylation of Dvl2.

### Comparison of PP5 and PP2A on Dvl2 dephosphorylation

**Figure 4:**
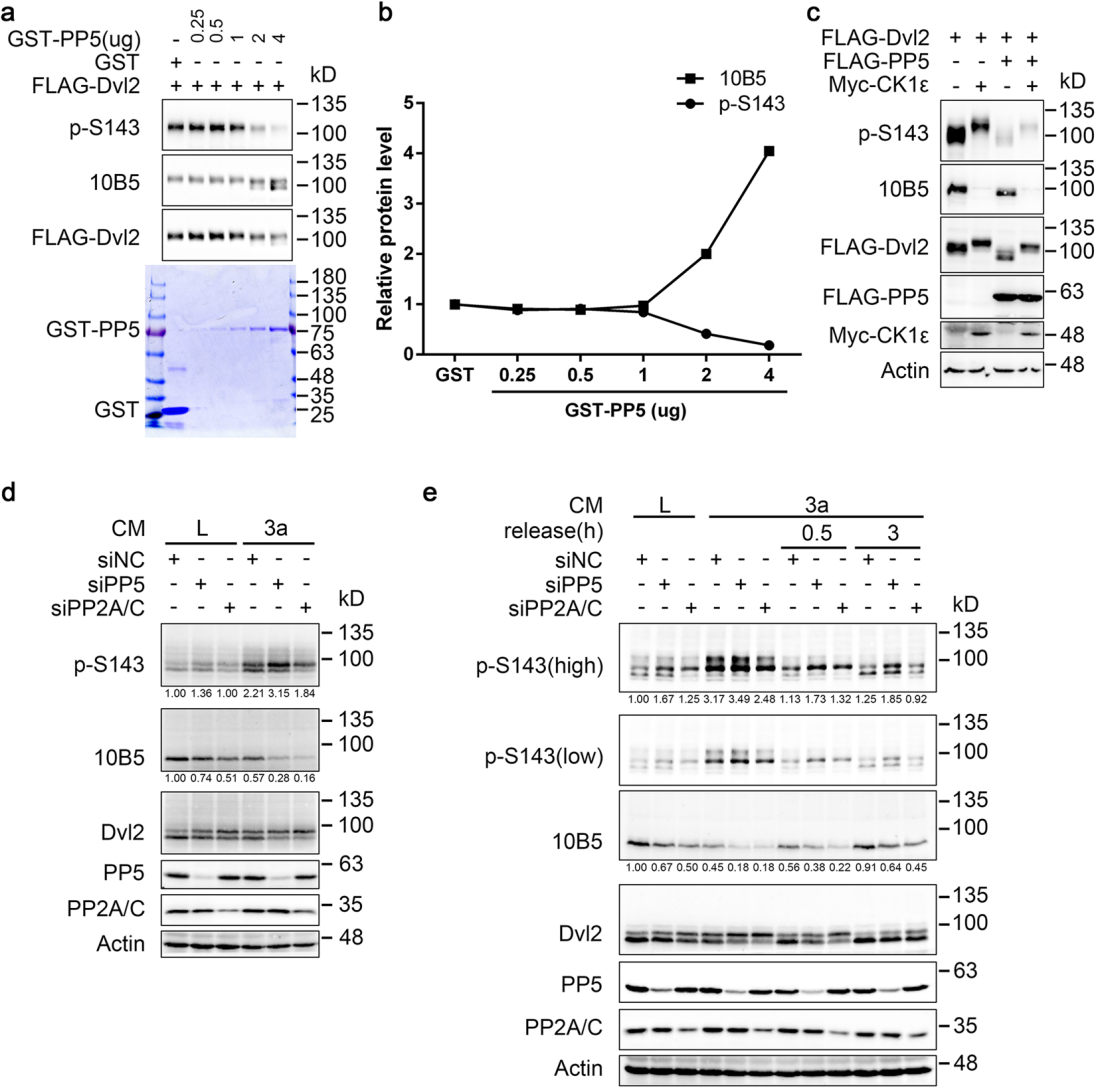
Comparison of PP5 and PP2A on Dvl2 dephosphorylation. (**a**) Highly phosphorylated FLAG-Dvl2 (coexpressed with Myc-CK1ε) was purified from transfected HEK293T cells using FLAG M2 beads and eluted by FLAG peptide. FLAG-Dvl2 protein was incubated with the indicated amounts of purified GST-PP5 protein for 1 h at 30 °C and then analysed by WB. Coomassie Brilliant Blue staining indicated the GST-PP5 and GST proteins added in the reactions. (**b**) Quantification of phosphorylation signal intensity from (**a**). The intensities of the phosphorylation signal and total Dvl2 signal from each sample were independently quantified and their ratio was calculated. The ratio from the GST-treated sample was set as 1 and was used to normalise the other samples. (**c**) HEK293T cells were transfected as indicated and were lysed 24 h later for WB analysis. (**d**) HCT116 cells were transfected with the indicated siRNA for 48 h, treated with L or Wnt3a CM for 1 h, and then the cell lysates were subjected to WB. Quantification of p-S143 and 10B5 signal intensity is shown below the bands. The intensities of the phosphorylation signal and total Dvl2 signal from each sample were independently quantified and their ratio was calculated. The ratio from L-CM-treated, siNC-transfected cells was set as 1 and was used to normalise the other samples. L, L CM (conditioned medium collected from control L-cells). 3a, Wnt3a CM. (**e**) HCT116 cells were transfected with the indicated siRNA for 48 h and then stimulated with L or Wnt3a CM for 2 h to induce Dvl2 phosphorylation. Cells were then washed with PBS, cultured in normal medium for the indicated time periods and subjected to WB. Quantification of p-S143 and 10B5 signal intensity is shown below the bands. The intensities of the phosphorylation signal and total Dvl2 signal from each sample were independently quantified and their ratio was calculated. The ratio from L-CM-treated, siNC-transfected cells was set as 1 and was used to normalise the other samples. L, L CM (conditioned medium collected from control L-cells). 3a, Wnt3a CM. Full-length gels and blots of this figure are shown in Figure S7.

The above results show that the effects of PP5 on the dephosphorylation of Dvl2 at the S143 and 10B5 sites differ to some degree. We therefore precisely compared the effect of PP5 on S143 and 10B5 sites *in vitro* and *in vivo*. In an *in vitro* phosphatase assay with increasing amounts of GST-PP5 applied, S143 and 10B5 sites were dephosphorylated with very similar efficiency, suggesting no preference *in vitro* (Fig. 4a,b). When coexpressed in HEK293T cells, PP5 could effectively dephosphorylate the S143 site in the presence of overexpressed CK1ε, which is responsible for the phosphorylation of several residues in Dvl2 including S143 and 10B5 sites^6,15^. However, under the same conditions, 10B5 sites were not dephosphorylated by PP5 (the 10B5 signal did not return when PP5 and CK1ε were coexpressed) (Fig. 4c). In other words, PP5 could counteract CK1ε at the S143 site but not at 10B5 sites when coexpressed in HEK293T cells. These findings suggest that the phosphorylated S143 site is more reactive to PP5 than 10B5 sites in HEK293T cells.

Next, we compared the involvement of PP5 and PP2A in the dephosphorylation of the S143 and 10B5 sites of Dvl2. It has been reported that PP2A interacts with and dephosphorylates Dvl2^31^. Indeed, siRNA-mediated knockdown of PP2A catalytic subunit (PP2A/C) led to an up-shift of Dvl2 protein, suggesting the overall enhancement of phosphorylation (Fig. 3a). However, when specific sites were carefully monitored in both untreated and Wnt3a-stimulated cells, it was observed that the enhancements of the Dvl2 protein up-shift and 10B5 phosphorylation (loss of 10B5 signal) were more dramatic in PP2A/C knockdown cells, whereas the elevation of S143 phosphorylation was more significant in PP5 knockdown cells (Fig. 4d). This is consistent with a previous report describing that phosphorylation of the 10B5 region contributes significantly to the protein up-shift phenomenon^15^. To further confirm this difference in a dynamic process, HCT116 cells were treated with Wnt3a CM to induce Dvl2 phosphorylation, followed by the washing out of Wnt3a to determine the dephosphorylation of Dvl2. In this process, again, PP2A/C knockdown resulted in more dramatic inhibition of 10B5 dephosphorylation, while PP5 knockdown mainly reduced S143 dephosphorylation (Fig. 4e). Together, these results suggested that, at least in HCT116 cells, PP5 and PP2A are not fully redundant in Dvl2 dephosphorylation.

### PP5 mutants affect Dvl2 subcellular localisation

The subcellular localisation of Dvl2 is regulated by many factors. In transfected cells, Dvl2 exists in two distinct patterns: diffuse and punctate^15^. Wnt ligand treatment or Fz coexpression can lead to the membrane recruitment of Dvl2^9,11,16^, while the coexpression of CK1ε promotes the diffuse pattern^16^. The phosphorylation of 10B5 sites has been shown to affect the subcellular localisation of Dvl2. Mutation of 10B5 sites into alanine leads to increased punctate localisation^15^. Our results suggest that PP5 can dephosphorylate Dvl2, so we then tested whether PP5 affects the subcellular localisation of Dvl2.

**Figure 5:**
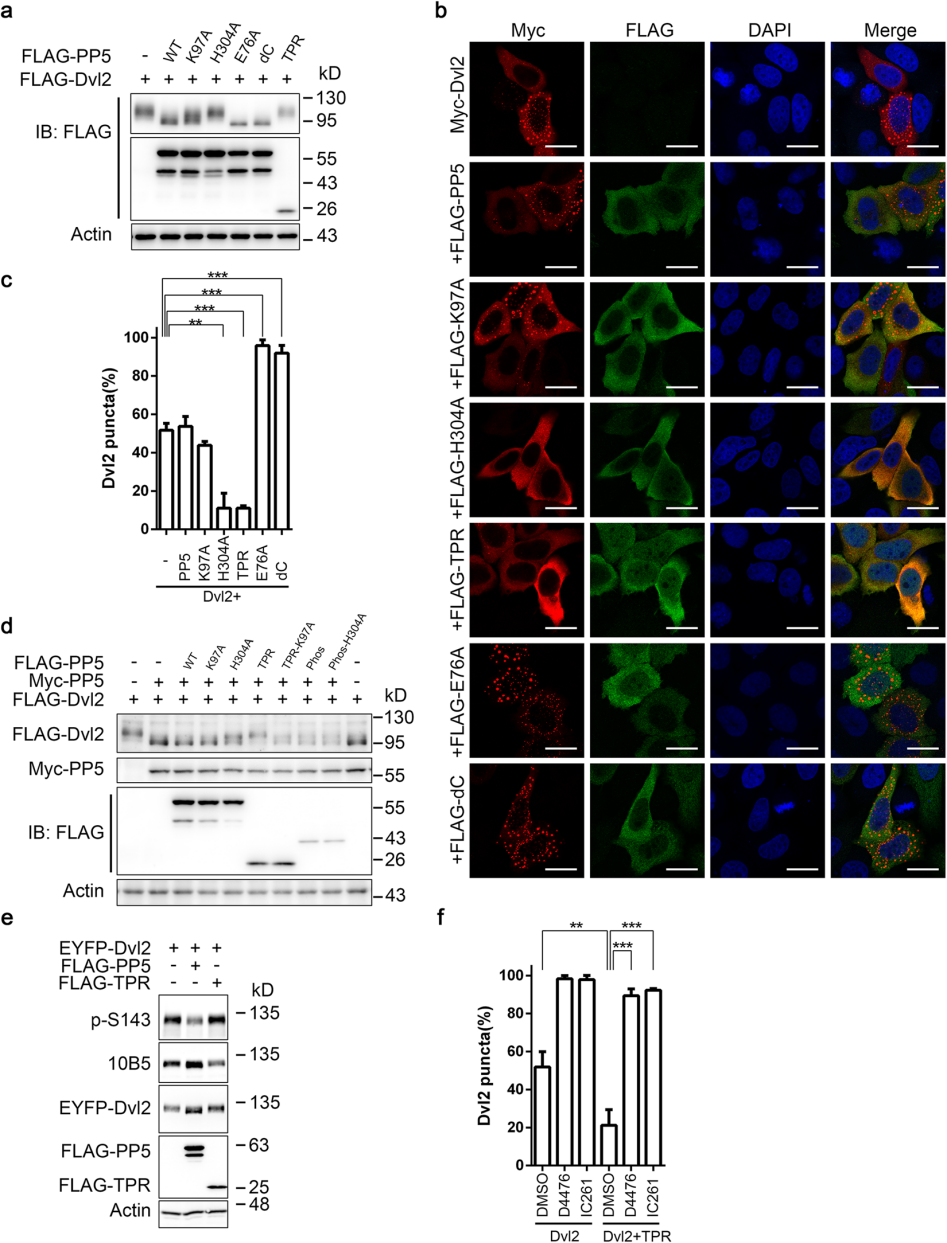
PP5 mutants affect Dvl2 subcellular localisation. (**a**) HEK293T cells were transfected with FLAG-tagged Dvl2 and wild-type or mutant PP5. Cells were lysed 24 h after transfection and subjected to WB. WT, wild-type PP5. K97A, PP5-K97A. H304A, PP5-H304A. E76A, PP5-E76A. dC, PP5-deltaC. (**b**) HeLa cells were transfected with Myc-Dvl2 alone or together with the FLAG-PP5 or PP5 mutants. Cells were stained 24 h after transfection with Myc (red) or FLAG (green) antibodies. Cell nuclei were stained with DAPI (blue). Scale bar, 20 μm. (**c**) Proportion of cells with Dvl2 puncta shown in (b). Results from three independent experiments were used for statistical analysis. Error bars, S.D. **p < 0.01, ***p < 0.001 (*t*-test). (**d**) HEK293T cells were transfected with FLAG-Dvl2, Myc-PP5 and a series of FLAG-tagged PP5 mutants. Cells were lysed 36 h after transfection and lysates were analysed by WB. The migration of FLAG-Dvl2 in gel was used as an indication of its degree of dephosphorylation. WT, wild-type PP5. K97A, PP5-K97A. H304A, PP5-H304A. (**e**) EYFP-Dvl2 was coexpressed in HEK293T cells with empty vector, FLAG-PP5 or FLAG-TPR. Cells were lysed 36 h later for WB analysis. (**f**) HeLa cells were transfected with Myc-Dvl2 alone or together with FLAG-TPR for 24 h and then treated with DMSO or CK1 inhibitors for 4 h as indicated. Cells were then stained as in (**b**). The proportion of cells with Dvl2 puncta is shown. D4476 was used at 50 μM and IC261 was used at 40 μM. Results from three independent experiments were used for statistical analysis. Error bars, S.D. **p < 0.01, ***p < 0.001 (*t*-test). Full-length blots of this figure are shown in Figure S8.

The phosphatase activity of wild-type PP5 is relatively low due to the autoinhibitory mechanism, which relies on the interaction between the N-terminal TPR domain and the C-terminal αJ helix. Two constitutively active PP5 mutants, PP5-E76A, which contains a critical amino acid mutation in the TPR domain disturbing αJ helix binding, and PP5-dC, which lacks amino acid residues critical for its C-terminal inhibitory domain^39,40^, were also utilised here to influence the subcellular localisation of Dvl2. We confirmed that both PP5-E76A and PP5-dC promoted the dramatic dephosphorylation of Dvl2 (Fig. 5a). In HeLa cells, wild-type PP5 or PP5-K97A had no dramatic influence on Dvl2 localisation. However, the constitutively active mutants PP5-E76A and PP5-dC dramatically increased the proportion of cells with Dvl2 in a punctate pattern. In contrast, coexpression with the phosphatase-inactive mutant PP5-H304A rendered Dvl2 mainly in a diffuse pattern in cells and, in fact, the TPR domain alone was sufficient to achieve this (Fig. 5b,c).

Considering that CK1ε-phosphorylated Dvl2 displays a diffuse pattern and 10B5 mutant Dvl2 tends to form puncta^15,16^, the PP5-E76A- and PP5-dC-induced punctuate distribution of Dvl2 was probably due to the dephosphorylation of 10B5 sites and potentially also some other sites with similar function. Regarding the TPR domain, it was previously suggested that this domain alone may behave as a dominant negative form capable of interfering with the function of full-length PP5^46–48^. We found that the TPR domain was capable of binding to Dvl2 (Fig. 2g) and could indeed block PP5-induced Dvl2 dephosphorylation (Fig. 5d). Similarly, the phosphatase-dead mutant PP5-H304A also blocked wild-type PP5-mediated Dvl2 dephosphorylation (Fig. 5d). In contrast, TPR-K97A, which showed reduced interaction with Dvl2 (Fig. 2g), had no significant effect on PP5-mediated Dvl2 dephosphorylation (Fig. 5d). Therefore, the effects observed here relied on the ability of the TPR domain to interact with Dvl2. This is consistent with the proposal that the TPR domain mediates protein–protein interaction^34^. Moreover, we found that coexpression of the TPR domain with Dvl2 caused hyperphosphorylation of the latter (reduced 10B5 signal) (Fig. 5e). Mechanistically, the effect of the TPR domain on Dvl2 subcellular localisation depended on endogenous CK1δ/ε family kinases, since this effect was reversed by their inhibitors D4476 and IC261 (Fig. 5f). Together, these results further demonstrate that PP5 can influence Dvl2 subcellular localisation via direct binding and regulating its phosphorylation.

### PP5 modulates Dvl2-mediated primary ciliogenesis

Apart from its well-known functions in Wnt signalling, Dvl2 has been shown to be important during the biogenesis of primary cilia^6,7,49–51^. Primary cilia are organelles that protrude out of eukaryotic cells and are dynamically assembled and disassembled during the cell cycle^52–54^. Dvl2 plays essential roles in multiple steps of ciliogenesis. In particular, S143-phosphorylated Dvl2 was detected at the centrosome and basal body of cilia. Phosphorylated S143, together with T224, creates a surface for interaction with Plk1 and subsequently the Dvl2/Plk1 complex promotes Aurora A-mediated disassembly of primary cilia^6^. Our results suggested that PP5 can dephosphorylate Dvl2, particularly at the S143 site. Therefore, we investigated whether PP5 functions in Dvl2-mediated ciliogenesis. hTERT-RPE1 cells, an hTERT-immortalised retinal pigmented epithelial cell line widely used in studies on cilium biogenesis, were applied here. In these cells, serum starvation can induce primary cilium formation and the re-addition of serum leads to cilium disassembly^55^.

**Figure 6:**
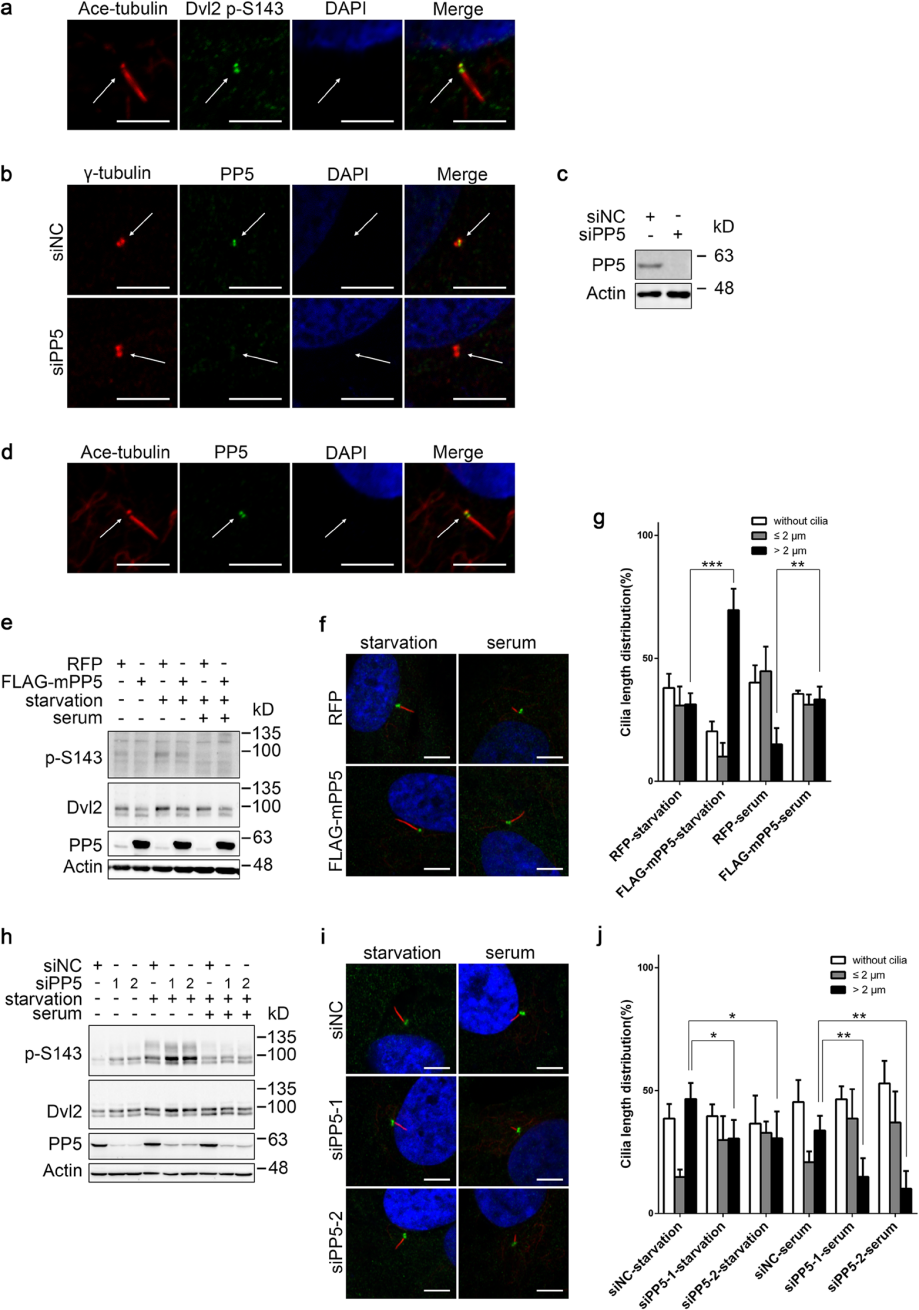
PP5 modulates Dvl2-mediated primary ciliogenesis. (**a**) Serum-starved hTERT-RPE1 cells were stained with Ace-tubulin (red) and Dvl2 p-S143 (green) antibodies. Cell nuclei were stained with DAPI (blue). Scale bar, 5 μm. (**b**) hTERT-RPE1 cells were transfected for two rounds with negative control (siNC) or PP5 siRNA (siPP5) and then stained with γ-tubulin (red) and PP5 (green) antibodies. Cell nuclei were stained with DAPI (blue). Scale bar, 5 μm. (**c**) The cells were transfected as in (**b**) and lysed for WB analysis. (**d**) Serum-starved hTERT-RPE1 cells were stained with Ace-tubulin (red) and PP5 (green) antibodies. Cell nuclei were stained with DAPI (blue). Scale bar, 5 μm. (**e**–**g**) hTERT-RPE1 cells stably expressing RFP (as a control) or FLAG-mPP5 were serum-starved for 48 h to induce the assembly of cilia. The disassembly of cilia was triggered by the addition of 10% serum for 3 h. These cells were then analysed by WB (**e**) or stained with Ace-tubulin antibody to show cilia (red), γ-tubulin antibody to show basal bodies (green) and DAPI to show cell nuclei (blue) (**f**). Scale bar, 5 μm. Proportions of cilia with different lengths are shown in (**g**). The lengths of cilia from cells in four randomly selected fields were measured; for each sample, about 150 cells were included. Error bars, S.D. **p < 0.01, ***p < 0.001 (*t*-test). (**h**–**j**) hTERT-RPE1 cells were transfected with control or PP5 siRNA. The cells were then treated and verified as in (**e**–**g**). Error bars, S.D. *p < 0.05, **p < 0.01 (*t*-test). Full-length blots of this figure are shown in Figure S9.

We first examined the subcellular distribution of Dvl2 and PP5 in hTERT-RPE1 cells. S143-phosphorylated Dvl2 was found at the centrosome^6^ and we confirmed this in hTERT-RPE1 cells (Fig. S3). Specifically, p-S143 signal was detected at the basal body of cilia (Fig. 6a), as previously reported^6^. PP5 was mostly observed in the cytoplasm and nucleus^33,37,42,56–58^. However, it was noted that overexpressed PP5 localised at the centrosome during mitosis in COS-1 cells^59^. To further clarify the localisation of PP5 in hTERT-RPE1 cells, we co-stained endogenous PP5 with the centrosome marker γ-tubulin, following an extraction and fixation protocol^6^. The results indicated clearly that endogenous PP5 protein was present at the centrosome in most of the cells (Fig. 6b). The signal was specific because siRNA-mediated knockdown diminished the staining (Fig. 6b). The efficiency of PP5 siRNA in hTERT-RPE1 cells was confirmed by Western blotting (Fig. 6c). Consistently, PP5 was also detected at the basal body of primary cilia in serum-starved cells (Fig. 6d). These results suggest that phosphatase PP5 is associated with centrosomes and basal bodies of cilia in cells.

Next, we examined the effects of PP5 overexpression or knockdown on Dvl2 S143 phosphorylation as well as the assembly and disassembly of cilia in hTERT-RPE1 cells. Overexpression of PP5 reduced Dvl2 S143 phosphorylation at the basal level, but also after serum starvation (Fig. 6e). Accordingly, serum-starvation-induced ciliogenesis was slightly enhanced, but more significantly the proportion of cells with longer primary cilia (>2μm) was increased in PP5-overexpressing cells (p < 0.001). In these cells, serum-addition-induced disassembly of cilia, particularly of the longer ones, was also delayed (p < 0.01, Fig. 6f,g). Conversely, in PP5 knockdown cells, Dvl2 S143 phosphorylation, especially after serum starvation, was enhanced (Fig. 6h). Consistent with this, serum-starvation-induced formation of cilia, especially the longer ones, was less abundant (p < 0.05). Upon serum re-addition, the disassembly of the long cilia also occurred more rapidly in PP5 knockdown cells than in control siRNA-treated cells (p < 0.01, Fig. 6i,j). We noted, however, that after serum addition, S143 phosphorylation of Dvl2 was rapidly abolished and this process was not significantly interfered by PP5 knockdown (Fig. 6h). This is probably due to PTEN making a more important contribution in this time period^7^. Nevertheless, the overall phenotype caused by the knockdown of PP5 was similar, to some extent, to that of PTEN knockdown. Taken together, our results indicate that PP5 is a phosphatase associated with centrosomes and basal bodies of cilia and plays a modest role in controlling the homeostasis of Dvl2 phosphorylation during ciliogenesis.

## Discussion

As an important cytoplasmic mediator of both canonical and noncanonical Wnt signalling, Dvl is extensively regulated by phosphorylation, which may affect its subcellular localisation, stability and activity toward different Wnt signalling branches^15,16,32,60^. More than 50 residues in Dvl were found to be phosphorylated under certain conditions and several kinases were shown to be involved^1,2^. However, the phosphatases responsible for Dvl dephosphorylation were not investigated as comprehensively as kinases. In the current study, we identified PP5 as a Dvl2 phosphatase and analysed its effects on Dvl2 phosphorylation, subcellular localisation and function.

To identify a Dvl2 phosphatase, an overexpression approach was adopted and a commercial plasmid library of human phosphatase genes was screened. The mobility shift of Dvl2 protein in SDS-PAGE was used as an indicator of its phosphorylation status. The fact that known Dvl2 phosphatases included in the library (PPP2CA and PPP2CB) were all identified suggested that the screen was sufficiently sensitive and provided confidence that the newly identified one (PPP5C) from this screen is probably as significant as the known ones. However, we noted that the strategy has some limitations. First, different phosphorylation sites have different effects on Dvl2 mobility. It was reported that the phosphorylation of some sites in the C-terminal of Dvl (including 10B5 sites) contributes greatly to the mobility shift of Dvl^15,16^. Therefore, phosphatases that are less effective at these sites would probably not show up in this screen. For example, PTEN dephosphorylates S143 of Dvl2, but does not affect its mobility when coexpressed^7^. Moreover, the phosphorylation of Dvl2 was highly regulated by extracellular stimulus and, in our screen, we verified only the basal level, without any stimulation. We might have missed those that function stringently under certain stimulations.

Although it was considered for a long time that phosphatases do not function as specifically as kinases, in terms of substrate and site selectivity, it has become increasingly evident that at least some of the phosphatases, including PP5, function in a site-specific manner^35,44,61,62^. For example, PP5 dephosphorylates Raf-1 at S338, but not as significantly at S259 or S621^61^. On Dvl2, the S143 site tends to be more affected by PP5 and 10B5 sites are slightly less so, in culture cells (Fig. 4c–e). Mass spectrometry also indicated that some of the sites were affected by overexpressed PP5, while others were not (Fig. 1e). This difference is consistent with the observation that the N-terminus of Dvl2 mediates PP5 interaction (Fig. 2h,i). In contrast, PP2A was found to bind the C-terminal DEP domain of Dvl2 and was more sufficient for 10B5 dephosphorylation (Fig. 4d,e)^31^. These results suggest that, although both PP2A and PP5 function as Dvl2 phosphatases, they are not fully redundant.

The primary cilium is a kind of organelle widely found on mammalian cells and functions in the sensing and integration of several important cellular signals. Ciliary defects lead to diseases termed ciliopathies^52,53^. The potential link between cilia and cancer was also raised recently^54^. Primary cilia emerge from the mother centriole, which transforms into basal body in the process of ciliogenesis. Several important regulators of ciliogenesis function at the basal body^53^. S143-phosphorylated Dvl2 was also found at the centrosome and basal body of cilia, together with CK1ε^6^. Our data demonstrated that PP5 strongly binds to CK1ε-phosphorylated Dvl2 and can very effectively counteract CK1ε-mediated Dvl2 phosphorylation (Figs 2e and 4c). Strikingly, PP5 was also detected at the centrosome as well as the basal body of cilia in hTERT-RPE1 cells (Fig. 6b–d). Our preliminary data indicated that the overexpression of PP5 enhanced while its knockdown reduced ciliogenesis, albeit modestly (Fig. 6g,j). Dvl2 is important for ciliogenesis, but its roles during this dynamic process are complex, ranging from apical docking of the basal body to the disassembly of cilia^6,7,49,50,63,64^. Its phosphorylation status is also highly dynamic^6,7,15,16,21,23,25,30,65^. Further investigation is needed to clarify Dvl2′s specific role in each step of ciliogenesis and when and how PP5 is involved.

## Methods

### Cell lines, reagents and antibodies

HEK293T, MCF7 and HeLa cells were cultured in Dulbecco’s modified Eagle’s medium (Macgene). HCT116 cells were cultured in McCoy’s 5A medium (Macgene). hTERT-RPE1 cells were cultured in DMEM/F12 medium (Gibco). Penicillin, streptomycin and 10% foetal bovine serum were added to all culture media unless otherwise noted. D4476 was purchased from Tocris Bioscience. IC261 was purchased from Selleckchem. Rabbit anti-Dvl2 and rabbit anti-PP2A/C antibodies were purchased from Cell Signaling Technology. Mouse anti-Dvl2 (10B5) and mouse anti-Myc antibodies were purchased from Santa Cruz Biotechnology. Rabbit anti-Dvl2 (phospho S143) and rabbit anti-γ-tubulin antibodies were purchased from Abcam. Mouse anti-PP5 antibody (for Western blotting) was purchased from BD Transduction Laboratories. Rabbit anti-PP5 antibody (for immunofluorescence) was purchased from Bethyl. Mouse anti-FLAG, mouse anti-acetylated tubulin and mouse anti-γ-tubulin antibodies were purchased from Sigma-Aldrich. Mouse anti-β-actin antibody was purchased from Sungene.

### Plasmids

The plasmid library of human phosphatase genes was purchased from Transomic Technologies (Cat. No. TCH3009). Full-length human Dvl2, PP5 and the truncated versions of them were cloned into the pCS2+ plasmid with N-terminal FLAG, Myc or EYFP tagging. Point mutants were constructed with the TransGen Easy Mutagenesis System. All constructs were confirmed by DNA sequencing. Plasmids were transfected into cells with VigoFect transfection reagent (Vigorous) for transient expression and with Lipofectamine 2000 (Invitrogen) for virus packaging.

### *In vitro* phosphatase assay

Wild-type and mutant PP5s were cloned into the pPGH vector. Upon transduction of BL21 bacteria, protein expression was induced by IPTG. After cell lysis, N-terminal GST-tagged PP5 proteins were purified with Glutathione Sepharose 4B beads (GE). For purification of FLAG-Dvl2 protein, HEK293T cells were transfected with pCS2+ FLAG-Dvl2 plasmids and lysed 36 h after transfection in RIPA buffer [10 mM Tris-HCl pH7.5, 150 mM NaCl, 0.5 mM EDTA, 0.1% SDS, 1% Triton X-100, 1% sodium deoxycholate, supplemented with protease inhibitor cocktail (Roche) and phosphatase inhibitor cocktail (Biotool)]. After centrifugation, FLAG-M2 beads (Sigma-Aldrich) were added to the supernatant and incubated for 4 h at 4 °C. After extensive washing, FLAG-Dvl2 protein was eluted with FLAG peptide. *In vitro* phosphatase assay was performed as described previously^61^.

### siRNA

Two independent siRNAs targeting human PP5 were designed and synthesised by RayBiotech. siRNA targeting PP2A/C was as described previously^66^. siRNA was transfected with Lipofectamine 2000 following the manufacturer’s instructions. The siRNA sequences were as follows.

siPP5-1: 5′-GAAGAAACTGCACCGGAAA-3′;

siPP5-2: 5′-CTATGACCTCCTCAACATA-3′;

siPP2A/C: 5′-ATGGAACTTGACGATACTCTA-3′.

### shRNA and stable lines

For shRNA-mediated PP5 knockdown, two independent shRNA constructs targeting human PP5 and a control vector were purchased from Sigma-Aldrich. For a stable cell line overexpressing mouse PP5, the coding sequence of mouse PP5 was amplified from mouse cDNA and inserted into the pLenti-BSD vector with an N-terminal FLAG tag. RFP inserted in the same vector was used as a control. Lentivirus was packaged in HEK293FT cells. After cell infection, puromycin or blasticidin was used for selection of stable cell lines.

### Western blotting, coimmunoprecipitation and GST pull-down

For Western blot analysis, cells were washed with ice-cold PBS and lysed in RIPA buffer freshly supplemented with protease inhibitors and phosphatase inhibitors. Cell lysate was denatured at 95 °C following the addition of SDS loading buffer. For coimmunoprecipitation with FLAG M2 beads or Myc beads (Abmart), cells were lysed in lysis buffer (20 mM Tris-HCl pH7.5, 150 mM NaCl, 2 mM EDTA, 1% NP-40, supplemented with protease inhibitor cocktail and phosphatase inhibitor cocktail). Coimmunoprecipitation with GFP-Trap beads (Chromotek) was performed following the manufacturer’s instructions. GST pull-down assay was performed as described previously^67^.

### Immunofluorescence

Immunofluorescence analysis was performed as described previously^68^. Briefly, cells seeded on coverslips were washed with PBS and fixed with 4% PFA for 15 min. After permeabilisation with 0.2% Triton for 10 min, cells were blocked with 2% BSA for 30 min. Then, cells were incubated with primary antibodies overnight at 4 °C. After three washes with PBS, cells were incubated with secondary antibodies for 1 h at room temperature. Nuclei were stained with DAPI. For the detection of primary cilia, cells were fixed with 4% PFA for 15 min followed by cold methanol permeabilisation for 10 min at −20 °C. The detection of Dvl2, Dvl2 p-S143 and PP5 localised at basal bodies was performed as described previously^6^. Briefly, cells were extracted for 30 s with extraction buffer (80 mM PIPES pH6.8, 0.5% Triton X-100, 1 mM MgCl_2_, 1 mM EGTA) followed by fixation with cold methanol. After washing with 0.1% Triton X-100 in PBS four times, cells were incubated with primary antibodies for 2 h at room temperature. After washing, cells were further incubated with secondary antibodies for 1 h at room temperature. Cell images were taken with a Zeiss LSM710META confocal microscope.

### MS analysis

HEK293T cells were transfected with EYFP-Dvl2 together with or without FLAG-PP5. Cells were lysed in RIPA buffer 36 h later. After centrifugation, the supernatant was collected and incubated with GFP-Trap beads for 4 h at 4 °C to purify EYFP-Dvl2 protein. The beads were extensively washed and samples were denatured at 95 °C following the addition of SDS loading buffer. Samples were separated with PAGE and stained with Coomassie Brilliant Blue. EYFP-Dvl2 bands were excised for MS analysis. Samples were sequentially digested with AspN and chymotrypsin. MS analysis of Dvl2 phosphorylation sites was performed as reported previously^16^. The phosphopeptides were further verified using the phosphoRS 3.1 node in Proteome Discoverer software, which determines the localisation of phosphorylation sites within validated peptide sequences. All MS/MS spectra corresponding to phosphopeptides were manually examined.

### Induction and disassembly of cilia

Induction and disassembly of primary cilia were performed as reported previously^6^. Briefly, hTERT-RPE1 cells were seeded on coverslips and, 24 h later, they were washed with PBS and starved in serum-free medium for 48 h. Disassembly of cilia was induced by the addition of 10% FBS for the indicated times.

### Statistical analysis

Data are presented as mean ± S.D. Two-tailed Student’s *t*-test was used to evaluate the statistical significance of differences in mean values between two populations (p < 0.05). The library screen (Fig. S1) was performed only once with individual positive clones retested. The MS analysis (Fig. 1e, Fig. S2 and Table S2) were performed twice with similar results. All other figures are representative of at least three independent experiments with similar results.

## Electronic supplementary material

Supplementary information
