# Supplementary material for: PP5 (PPP5C) is a phosphatase of Dvl2
Source: Sci Rep. 2018 Feb 9;8:2715. doi: 10.1038/s41598-018-21124-3 (PMC5807433; doi:10.1038/s41598-018-21124-3)

Supplementary information

PP5 (PPP5C) is a phosphatase of Dvl2

Jianlei Xie[1], Meng Han[2], Miaojun Zhang[1], Haiteng Deng[2], Wei Wu[1*]

[1]MOE Key Laboratory of Protein Science, School of Life Sciences, Tsinghua University, Beijing, 100084, China

[2]MOE Key Laboratory of Bioinformatics, School of Life Sciences, Tsinghua University, Beijing, 100084, China

*Correspondence: WW, wwu@mail.tsinghua.edu.cn

Supplementary Figure legends

Table S1. Contents of the plasmid library of human phosphatase genes.

Table S2. Phosphorylation sites identified by mass spectrometry analysis.

The intensity values of peptides containing the listed phosphorylation sites are shown. The ratio was calculated as follows: intensity of phosphopeptide / (intensity of phosphopeptide + intensity of nonphosphopeptide).

Figure S1. Identification of Dvl2 phosphatases.

FLAG-Dvl2 was transfected into HEK293T cells together with empty vector or plasmids from a human phosphatase library. Cell lysates were prepared 48h later and subjected to Western blot analysis using FLAG antibody. The appearance of more rapidly migrating bands was used as an indication of dephosphorylation (arrowhead).

Figure S2. Mass spectrometry analysis of Dvl2 phosphorylation.

(**a**), EYFP-Dvl2 was transfected alone or together with FLAG-PP5 into HEK293T cells and, 36h later, cell lysates were prepared. EYFP-Dvl2 protein was purified with GFP-Trap beads and stained with Coomassie Brilliant Blue after separation by SDS-PAGE. (**b**), The Dvl2 bands in (a) were excised and analysed by mass spectrometry. The y and b series indicate fragments at amide bonds of the peptide. Only S143 phosphopeptide is shown here as a representative.

Figure S3. S143-phosphorylated Dvl2 is detected at the centrosome in hTERT-RPE1 cells.

Serum-starved hTERT-RPE1 cells were stained with γ-tubulin (red) and Dvl2 p-S143 (green) antibodies. Cell nuclei were stained with DAPI (blue). Scale bar, 5 μm.

Figures S4–9. Full-length blots in this study.

Plate	Position	Symbol	Plate	Position	Symbol	Plate	Position	Symbol	Plate	Position	Symbol	Plate	Position	Symbol
1	A1	NT5C3	1	C1	PPP5C	1	E1	PTPN11	1	G1	PTPN4	2	A1	TENC1
1	A2	UBLCP1	1	C2	STYX	1	E2	INPP1	1	G2	PPM1D	2	B1	MTMR3
1	A3	LCK	1	C3	HDHD1	1	E3	PFKFB4	1	G3	ACP6	2	C1	PTPRU
1	A4	PPIP5K2	1	C4	FBP1	1	E4	PHPT1	1	G4	PPP3CA	2	D1	MTMR3
1	A5	DUSP10	1	C5	PPP2CA	1	E5	PPP3CB	1	G5	LPIN1	2	E1	TENC1
1	A6	PPM1K	1	C6	MTMR4	1	E6	CILP	1	G6	ACPL2	2	F1	VPS29
1	A7	MTMR14	1	C7	RPAP2	1	E7	PPM1D	1	G7	PTPRF	2	G1	SACM1L
1	A8	DUSP16	1	C8	ATP1A1	1	E8	MTMR12	1	G8	MTMR2	2	H1	PTPN9
1	A9	INPP5B	1	C9	PSPH	1	E9	CTDP1	1	G9	PTPDC1	2	A2	PTPN2
1	A10	NT5E	1	C10	PPP1R15B	1	E10	INPP5E	1	G10	ENOPH1	2	B2	MTMR8
1	A11	PPM1J	1	C11	PPP2R2D	1	E11	PHOSPHO2	1	G11	ACP5			
1	A12	PTPRR	1	C12	DUSP8	1	E12	ALPL	1	G12	ALPL			
1	B1	PGAM5	1	D1	NT5C	1	F1	PTPN1	1	H1	DLGAP5			
1	B2	PPM1M	1	D2	BPGM	1	F2	NT5C3L	1	H2	RNGTT			
1	B3	PPP2CB	1	D3	NT5C3L	1	F3	ALPP	1	H3	PPM1A			
1	B4	PTPRA	1	D4	DAPP1	1	F4	PPP3R1	1	H4	DUSP6			
1	B5	CCDC155	1	D5	HDHD3	1	F5	PDP2	1	H5	VPS29			
1	B6	PNKP	1	D6	MINPP1	1	F6	DUSP3	1	H6	CTDSPL2			
1	B7	NT5M	1	D7	MTMR6	1	F7	EYA3	1	H7	PPAP2A			
1	B8	PPIP5K1	1	D8	PPM1A	1	F8	MTMR14	1	H8	PPIP5K1			
1	B9	PTPN5	1	D9	PPP2R3B	1	F9	PPM1B	1	H9	CTDSP2			
1	B10	CDKN3	1	D10	PDXP	1	F10	PPM1N	1	H10	PTPN22			
1	B11	PGAM1	1	D11	ACP1	1	F11	INPP1	1	H11	LHPP			
1	B12	INPP4B	1	D12	PHLPP1	1	F12	PPAPDC1B	1	H12	APTX			

	EYFP-Dvl2			EYFP-Dvl2+FLAG-PP5				
site	intensity of phospho-peptide	intensity of non-phospho-peptide	ratio of phosphorylation	intensity of phospho-peptide	intensity of non-phospho-peptide	ratio of phosphorylation	+PP5/-PP5	change
S143	1.70E+09	7.86E+10	0.0212	3.59E+08	9.90E+10	0.0036	0.1704	down
S158	3.10E+10	6.21E+10	0.3330	8.28E+09	7.54E+10	0.0989	0.2970	down
S170	5.21E+07	7.13E+07	0.4220	0	4.43E+07	0	0	down
S194	6.54E+09	7.37E+09	0.4701	4.71E+09	8.61E+09	0.3535	0.7521	not significant
S207	3.06E+07	9.45E+08	0.0313	0	2.19E+09	0	0	down
S252	3.57E+08	0	1	0	1.57E+09	0	0	down
S358	1.57E+08	4.21E+10	0.0037	0	4.83E+10	0	0	down
S520	1.44E+09	3.88E+10	0.0357	6.81E+07	4.43E+10	0.0015	0.0430	down
S562	1.67E+10	3.34E+08	0.9804	9.98E+09	1.50E+08	0.9852	1.0048	not significant
S618	0	2.15E+09	0	8.36E+07	3.43E+08	0.1961	*	up
S717	0	1.61E+11	0	2.98E+09	1.82E+11	0.0161	*	up
S720	7.97E+09	1.29E+11	0.0582	0	1.37E+11	0	0	down

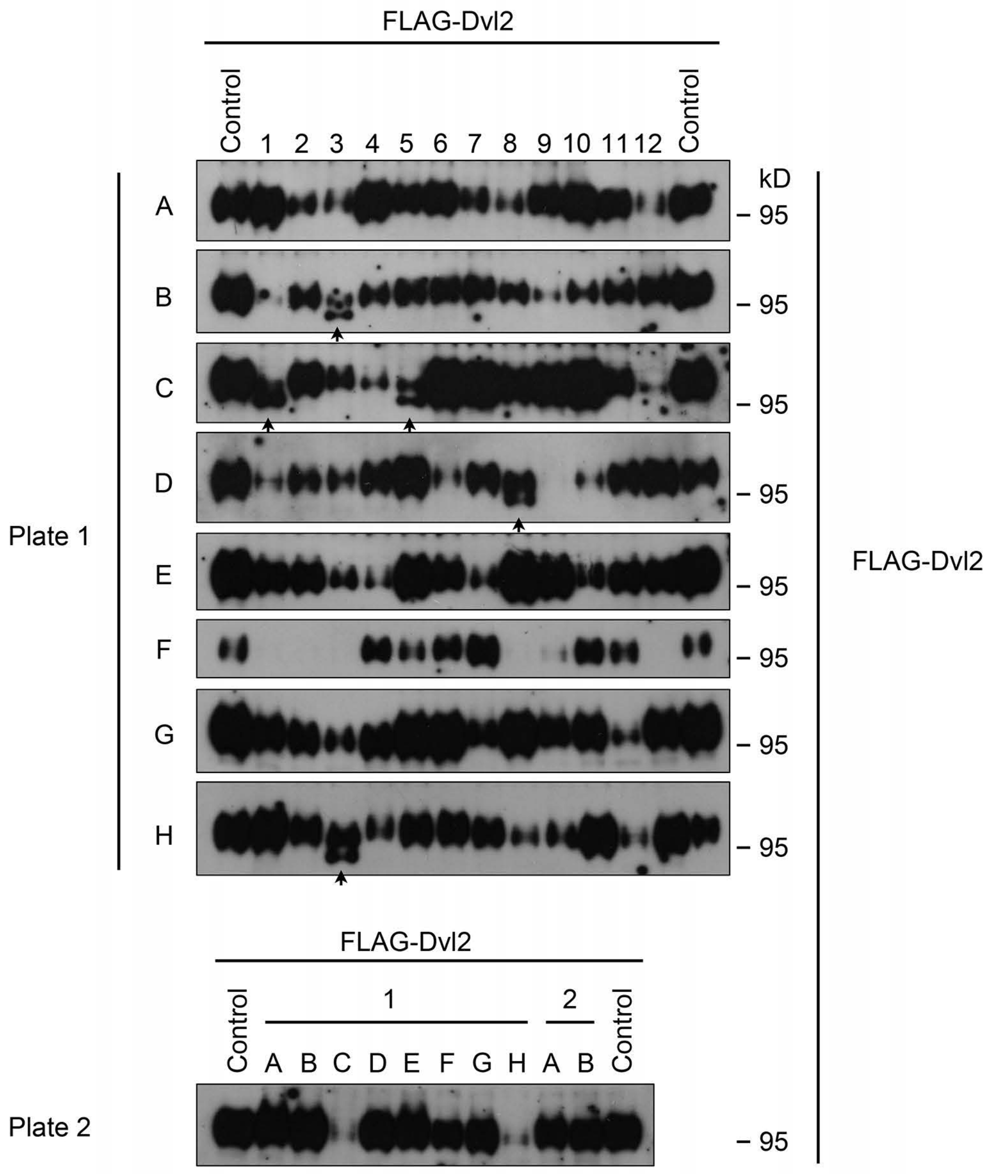
FLAG-Dvl2
Control
1 2 3 4 5 6 7 8 9 10 11 12
Control
kD
A
B
C
D
E
F
G
H
95
Plate 1
FLAG-Dvl2
FLAG-Dvl2
Control
1
2
A B C D E F G H A B
Control
Plate 2
95

Xie et al., 2018. Figure S2

a

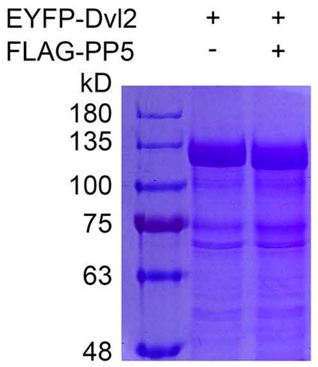

b

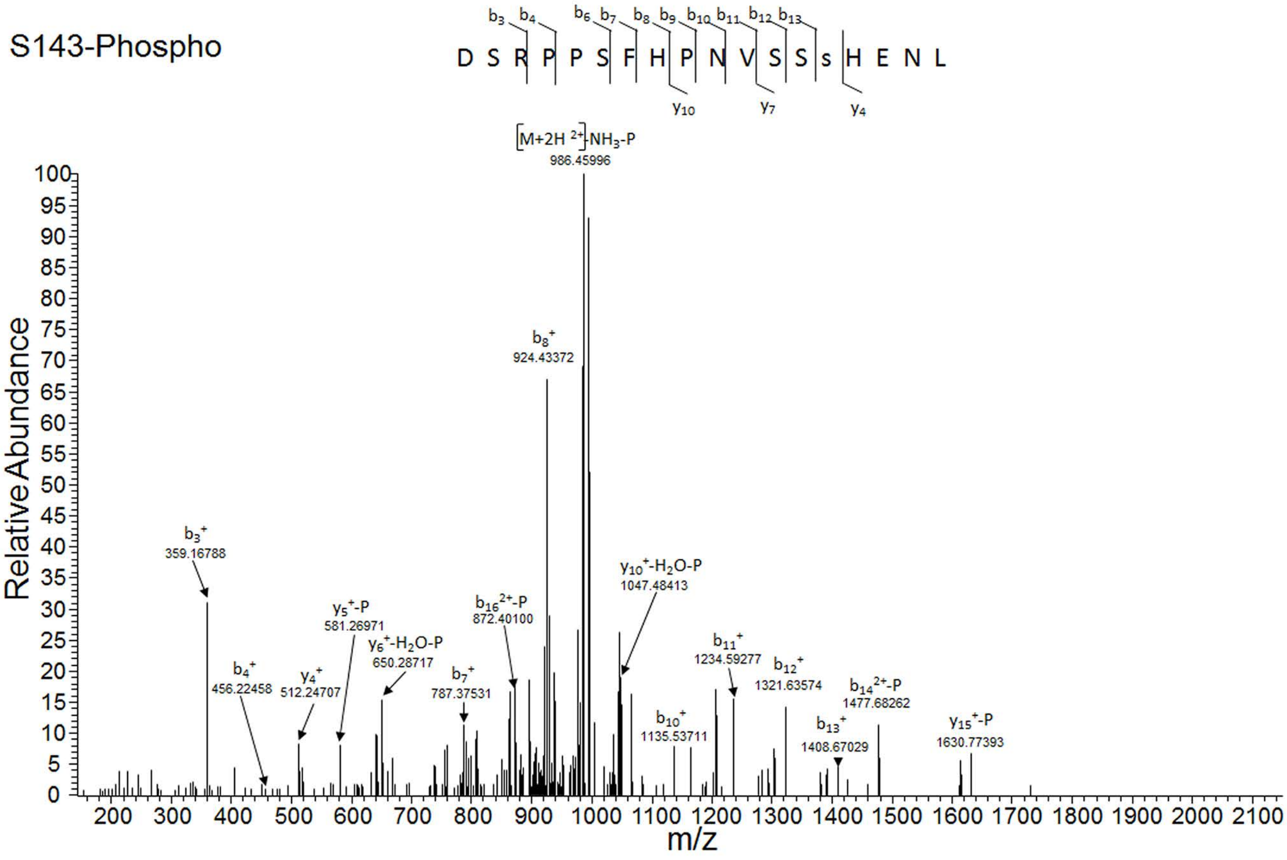

Xie et al., 2018. Figure S3

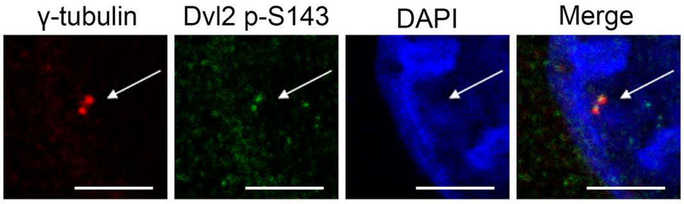

Xie et al., 2018. Figure S4

Fig. 1a

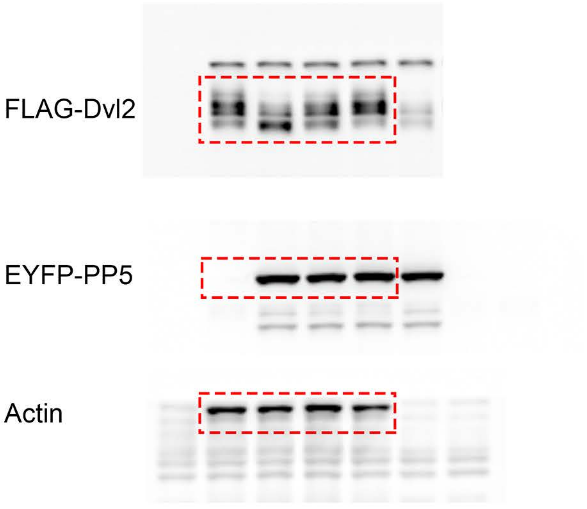

Fig. 1b

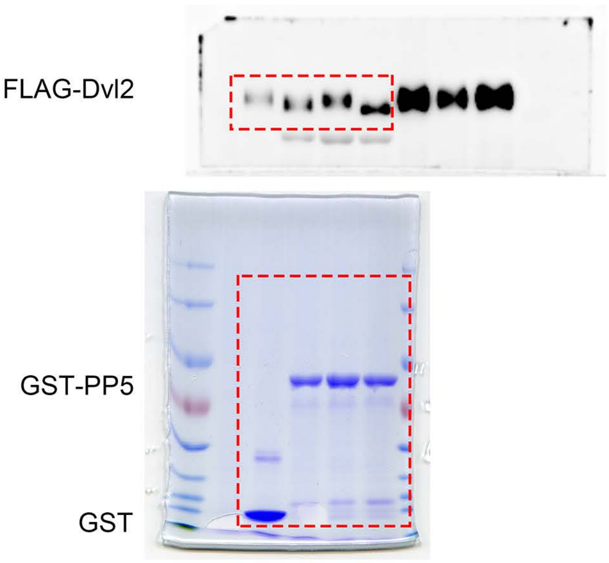

Fig. 1c

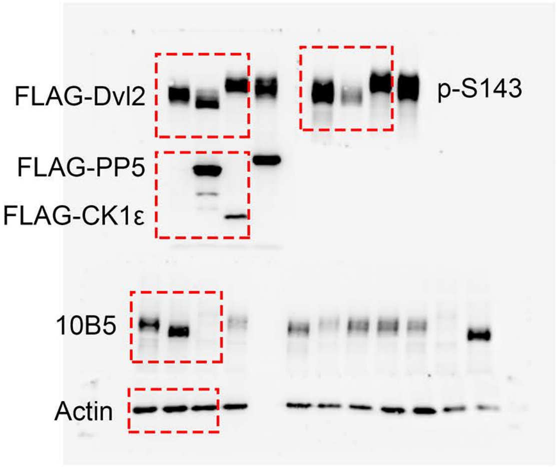

Fig. 1d

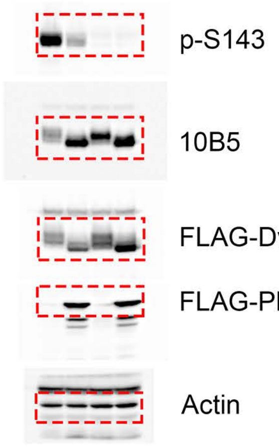

Xie et al., 2018. Figure S5

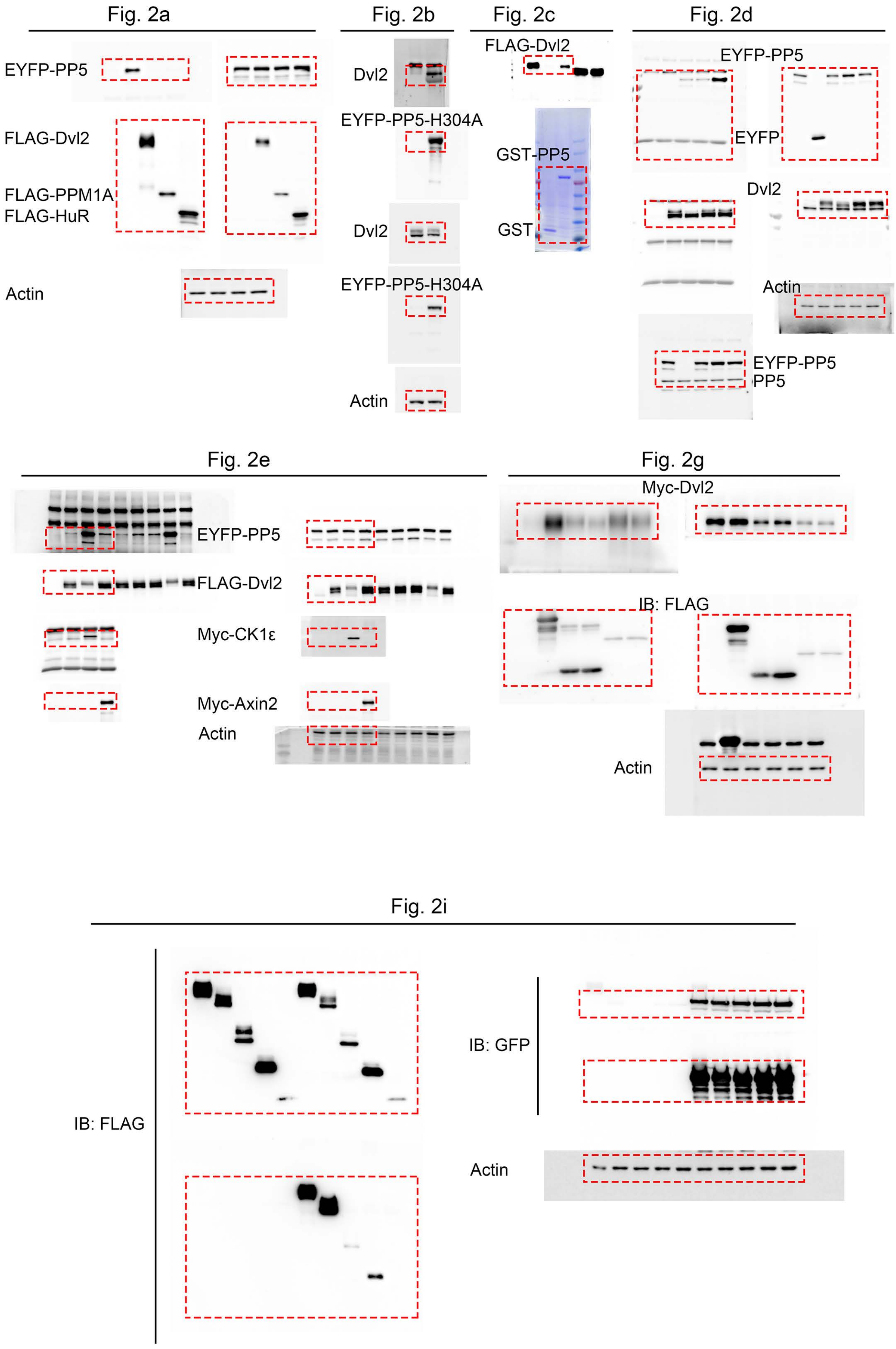

Xie et al., 2018. Figure S6

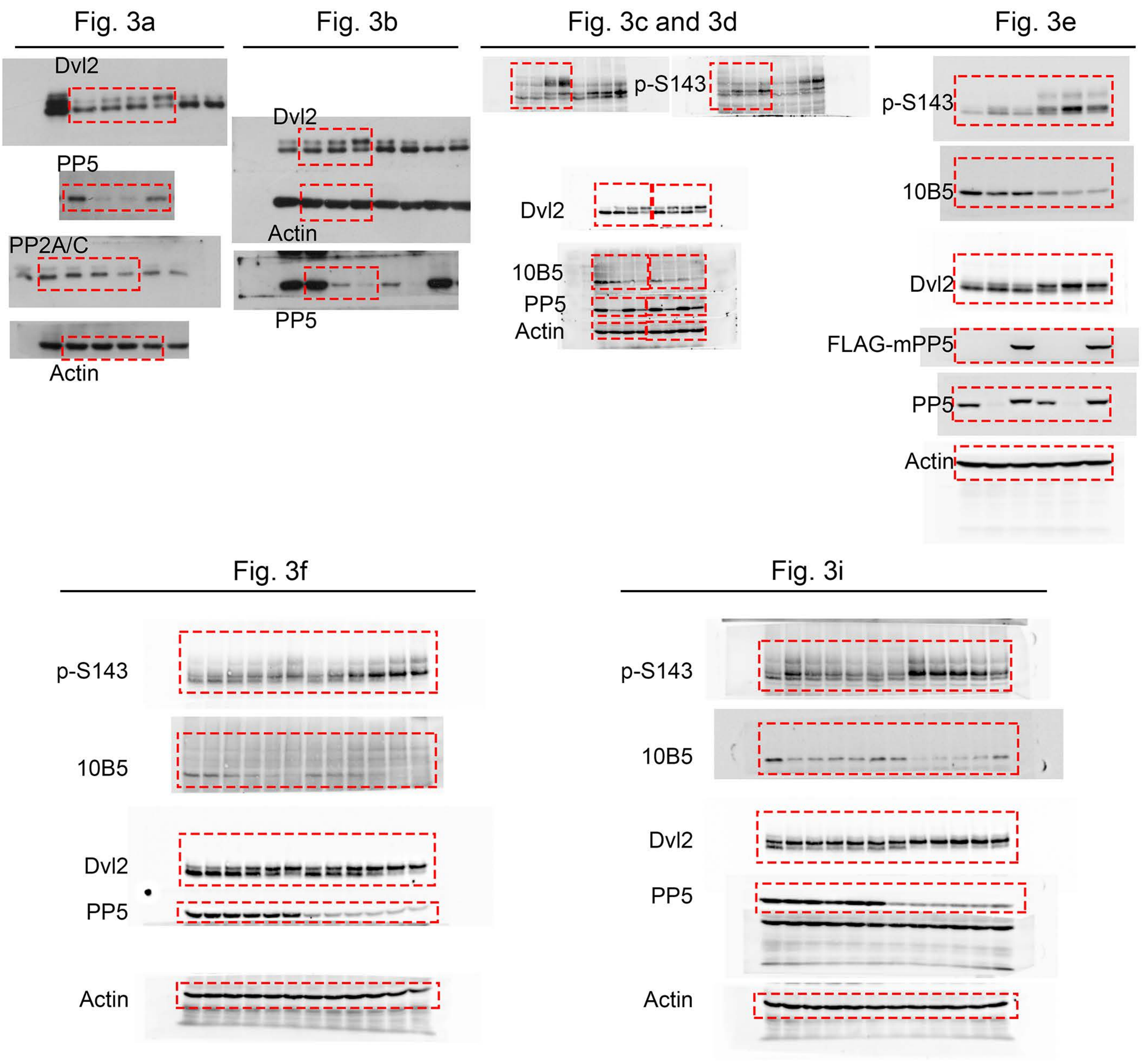

Xie et al., 2018. Figure S7

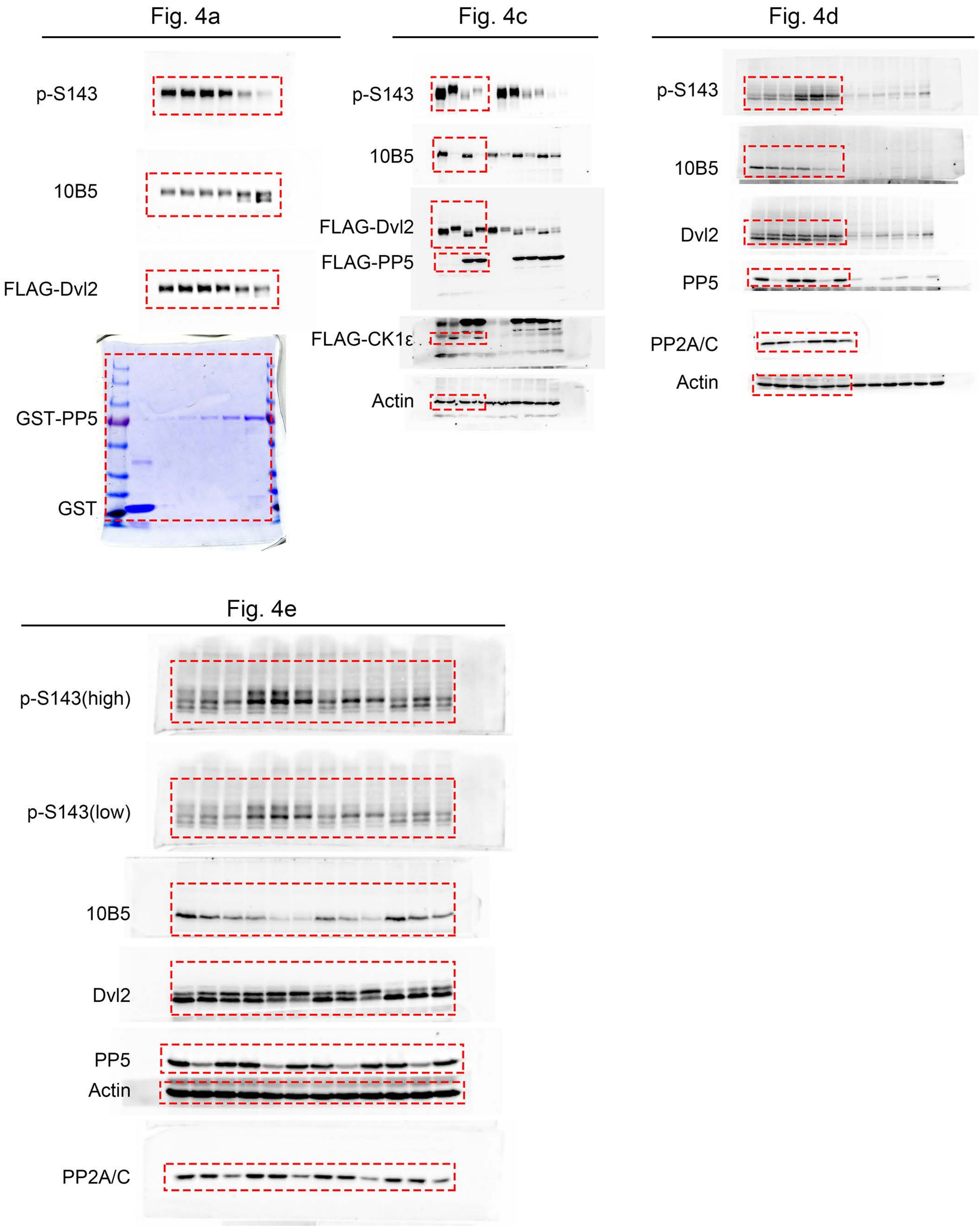

Xie et al., 2018. Figure S8

Fig. 5a

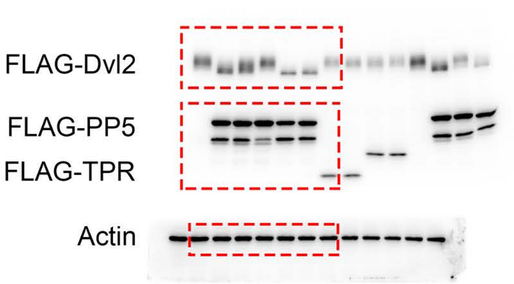

Fig. 5d

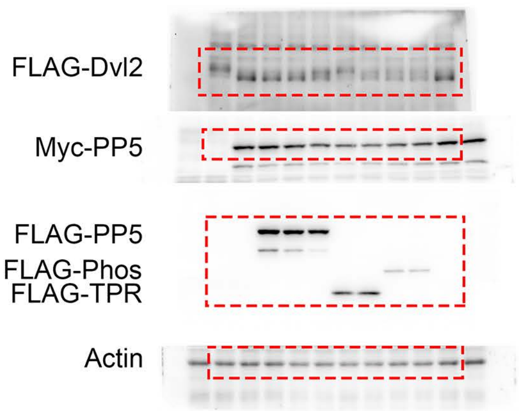

Fig. 5e

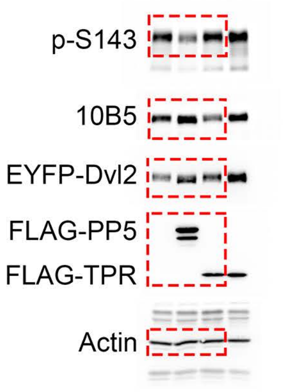

Xie et al., 2018. Figure S9

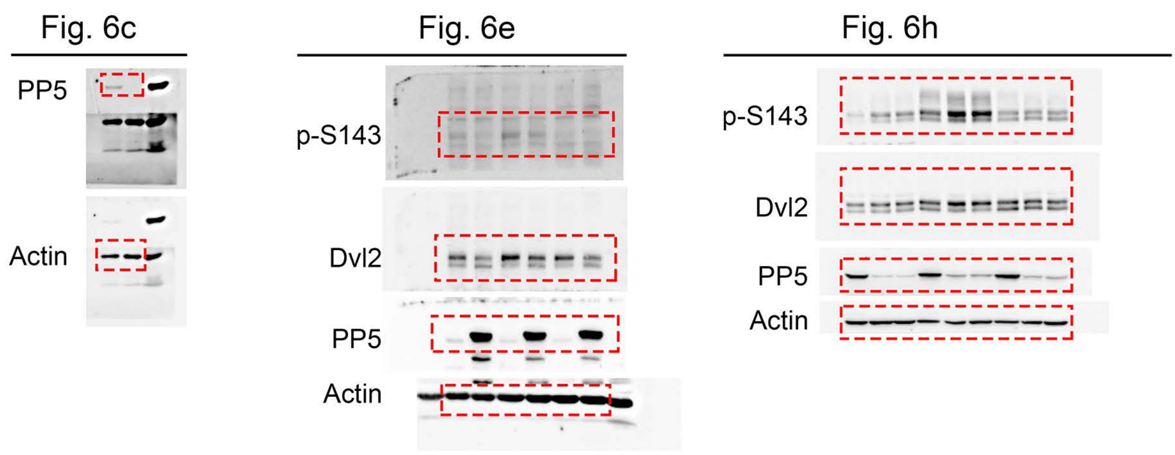

Supplement: Supplementary file 1 — Supplementary information [file 41598_2018_21124_MOESM1_ESM.pdf]
